# Effects of 15-Day Head-Down Bed Rest on Emotional Time Perception

**DOI:** 10.3389/fpsyg.2021.732362

**Published:** 2021-12-22

**Authors:** Yiming Qian, Shan Jiang, Xiaolu Jing, Yusheng Shi, Haibo Qin, Bingmu Xin, Lizhong Chi, Bin Wu

**Affiliations:** ^1^School of Psychology, Beijing Sport University, Beijing, China; ^2^China Astronaut Research and Training Center, Beijing, China; ^3^Engineering Research Center of Human Circadian Rhythm and Sleep (Shenzhen), Space Science and Technology Institute (Shenzhen), Shenzhen, China

**Keywords:** time perception, emotional stimuli, head-down bed rest, embodiment, behavioral imitation

## Abstract

Accurate time perception is clearly essential for the successful implementation of space missions. To elucidate the effect of microgravity on time perception, we used three emotional picture stimuli: neutral, fear, and disgust, in combination with a temporal bisection task to measure 16 male participants’ time perception in 15 days of –6° head-down bed rest, which is a reliable simulation model for most physiological effects of spaceflight. We found that: (1) participants showed temporal overestimation of the fear stimuli in the middle phase (day 8), suggesting that when participants’ behavioral simulations were consistent with the action implications of the emotional stimuli, they could still elicit an overestimation of time even if the subjective arousal of the emotional stimuli was not high. (2) Participants’ temporal sensitivity tends to get worse in the bed rest phase (days 8 and 15) and better in the post-bed rest phase, especially for neutral and fear stimuli, suggesting that multiple presentations of short-term emotional stimuli may also lead to a lack of affective effects. This reduced the pacemaker rate and affected temporal perceptual sensitivity. Also, this may be related to changes in physiological factors in participants in the bed rest state, such as reduced vagal excitability. These results provide new evidence to support the theory of embodied cognition in the context of time perception in head-down bed rest and suggest important perspectives for future perception science research in special environments such as microgravity.

## Introduction

Time perception is an important ability in everyday life and can influence individuals’ behavioral choices (Allman et al., 2014). For astronauts, correct time perception has an impact on flight space navigation performance and the execution of tasks according to the spaceflight schedule during space missions. When time perception is off, it may lead to improper manipulation or failure to complete tasks within the scheduled time (Christensen and Talbot, 1986; Zhang and Bai, 1999). Some early studies have confirmed that astronauts’ perception of time changes during space missions. Ratino et al. (1988) have found that astronauts overestimated the shortest duration task as the mission proceeds and had statistical significance in comparing preflight and postflight baselines. Based on the temporal control study of the two astronauts, Semjen et al. (1998) further concluded that during the space flight, the function of the internal timing module may change, so the regularity of motor timing is slightly damaged. However, due to the limitation of the published literature and the difficulty of copying the microgravity environment in space, we still know very little about the changes in time perception in microgravity.

When individuals are exposed to emotion-filled stimuli, they exhibit distorted temporal judgments, a phenomenon known as the time-emotion paradox (Droit-Volet and Gil, 2009), which has been confirmed by several empirical studies (Droit-Volet and Meck, 2007; Droit-Volet et al., 2016, 2020; Lake et al., 2016; Ghaheri et al., 2019). When astronauts live and work in isolated, confined and hostile environments like deep space for long periods of time, they are susceptible to negative emotions due to various types of stressors (Ishizaki et al., 2002; Chen et al., 2015; Brauns et al., 2019). It is therefore likely that, in addition to the weightless environment, changes in the astronauts’ perception of time in the space environment are related to emotion.

The internal clock model is the most classical explanation of interval timing perception (Treisman, 1963; Gibbon et al., 1984; Zakay and Block, 1996). The internal clock is composed of a pacemaker that emits pulses at a regular rate, a switch that controls the start and the stop of timing, and an accumulator that receives the pulses sent by the switch. Time perception is related to the number of pulses accumulated in the accumulator. On the one hand, arousal can accelerate the rate of pulses generated by the pacemaker, resulting in more pulses being collected by the accumulator, thus prolonging time perception (Droit-Volet et al., 2004; Effron et al., 2006). On the other hand, attention controls switch latency and the closure state of the switch. More attention is given, more pulses would be accumulated and the perceived duration is extended (Yarrow et al., 2004; Buhusi and Meck, 2009; Ann et al., 2011; Grommet et al., 2011; Au et al., 2012).

Researchers differ on how emotion distorts duration perception. These differences reflect the arguments between conceptual views of emotions (dimensional and embodiment emotions) in different cognitive frameworks (Lina et al., 2015). Dimensional emotion perspective emphasizes emotions to be composed of arousal and valence dimensions, the interaction of these two dimensions determining the perceptual and behavioral functions of emotions (Russell, 1980; Smith and Ellsworth, 1985). Studies supporting this perspective have concluded that emotional valence and arousal as the main factors influencing temporal judgments. For example, for high-arousal stimuli, the duration of negative pictures was judged longer than that of positive pictures. In contrast, for low-arousal stimuli, negative pictures were perceived as shorter in duration compared to positive pictures (Angrilli et al., 1997). According to this view, researchers have suggested that emotional stimuli with the same arousal and valence would produce similar temporal distortions, such that all negative high arousal stimuli would have a prolonged subjective time perception. However, studies supporting embodiment emotions have found contradictory results. The embodied cognitive view emphasizes the important role of the body in sensory-motor processes and considers cognition, the body and the environment as one (Clark, 1999; Niedenthal et al., 2005). For example, Gil and Droit-Volet (2011) asked participants to estimate the temporal distance of pictures of emotional faces. Both disgusted and angry faces elicited high arousal and unpleasantness, and the results showed that the duration of angry faces was estimated to longer relative to neutral faces, whereas the duration of disgusted faces was not affected. Similar result was found in Shi et al.’s (2012) study, where participants showed an overestimation of time with the threat stimulus and no effect with the disgust stimulus. This suggests that a single emotional dimension is not the key determinants of subjective timing. Different types of emotion be associated with different behavioral functions. Previous research has suggested that although both fear and disgust are classified as high arousal negative emotions, they activate different processes. Threat-related stimulus (e.g., the sight of a snake attack) activates our defensive system and biases the motor response (Bradley et al., 2001). Given that threatening or fearful events are most likely to be directed at our bodies (e.g., the sight of a snake attack), the link between what we see and what our bodies feel can be quickly established and prepared for action (Poliakoff et al., 2007). In contrast, disgust is more related to avoiding something that is harmful to our health or tastes bad (Rozin and Fallon, 1987), and the perception of disgust does not lead to a preparation for immediate action enabling the organism to avoid imminent danger (Gil and Droit-Volet, 2011). As a result, arousal levels do not increase and the internal clock does not speed up. In summary, embodiment emotion perspective suggests that imitation and potential action response elicited by emotional stimuli are the key determinants of subjective timing (Effron et al., 2006; Wittmann et al., 2010; Nather et al., 2011).

Head-down bed rest (HDBR) is widely accepted as a reliable model for simulating the physiological responses to weightlessness that occurs in space flight (Pavy-Le Traon et al., 2007; Pandiarajan and Hargens, 2020). Participants are placed in the supine position on a bed that is tilted 6 degrees to place the head closer to the ground and to elevate the feet. This restriction on body state is used to simulate physical deconditioning and cephalic fluid shifts during standard space missions (Hargens and Vico, 2016). Six-degrees is the international standard for simulating weightlessness (Smith et al., 2011). Some researchers have investigated the physiological cognitive functional changes during bed rest, such as affective processing (Brauns et al., 2019), memory and executive function (Shehab et al., 1998; Zhao et al., 2011; Liu et al., 2012), cooperation and aggression (Wang et al., 2017), judgment and decision making (Jiang et al., 2013; Rao et al., 2014). While according to the authors’ knowledge, no study has investigated the effects of long-duration bed rest on time perception.

Therefore, the present study used three emotional picture stimuli: neutral, fear, and disgust, in combination with a temporal bisection task to measure participants’ time perception in a 15-day –6° head-down bed rest. The aim was to reveal the effect of head-down bed rest on time perception and trends in change. We hypothesized that during head-down bed rest, consistent with the embodiment emotion perspective, fear stimuli elicit simulated and potential action responses that influence perceived duration, whereas disgust stimuli did not.

## Materials and Methods

### Participants

Data was collected from 17 male participants (mean age = 27 years, *SD* = 3; mean height = 168 cm, *SD* = 3.5; mean weight = 62.2 kg, *SD* = 5.8). Participants were recruited openly for this study. All participants were healthy, right-handed, had no smoking or drinking habits, had normal or corrected-to-normal vision, and no participants had a history of neurological or psychiatric disorders. No recent negative life events such as marital frustration or family changes. Written informed consent was obtained before the experiment and payment was received after the experiment.

### Materials

#### Emotion Pictures Stimuli

Three types of pictures were selected from the IAPS: fear stimuli evoking high-arousal (such as a snake, shark); disgust stimuli also classed as high on arousal (such as mutilation, burn victim); and neutral stimuli rated “neutral” in both valence and arousal. Fifty-five participants completed ratings of 58 emotional pictures and selected 30 pictures that met the valence and arousal criteria as the final stimulus materials. The final fear and disgust stimuli used had significantly lower valence (all *p* < 0.001) and higher arousal than the neutral stimuli (all *p* < 0.001), with no difference between the previous two (*p* > 0.05).

#### Positive Affect and Negative Affect Scale

The Positive Affect and Negative Affect Scale (PANAS) is comprised of 20 items and includes two emotional dimensions: positive and negative. The Chinese version of the PANAS has well-established validity and reliability (Qiu et al., 2008). Ten items were used to evaluate negative affect and the remaining ten items were used to evaluate positive affect. The raw score for each item ranged from one to five. Before the temporal bisection task, participants were required to complete this scale. The Cronbach’s α coefficients for each of the four tests in this study were 0.834, 0.904, 0.867, and 0.892, respectively.

### Experimental Procedure and Flow

The current bed rest experiment lasted for a total of 29 days, which comprised a pre-HDBR period of 7 days, a –6° HDBR period of 15 days to simulate the physiological effects of exposure to weightlessness and a post-HDBR recovery period of 7 days. During the 15 days bed rest period, participants were restricted to absolute head-down recumbence even while eating, excreting, bathing, and sleeping without pillows. They were freely permitted to watch videos, play games like Rubik’s Cube, read books and so on. Except for one participant, 2 participants were housed together in each room, with beds separated by moveable curtains. And the room temperature was kept at a comfortable temperature of approximately 25°C. Participants’ diets were provided in accordance with the standards set by the nutritionist and there were no restrictions on drinking water. During the HDBR period, physicians monitored the physical conditions of the participants, including taking measurements of the blood pressure, heart rate, breathing rate, body temperature, water consumption, urinary output, and general health condition of each participant. The daily schedule was as follows: awakening at 07:00 a.m., breakfast at 07:30–8:00 a.m., lunch at 11:30–12:00 a.m., afternoon nap at 12:00–13:00 p.m. and dinner at 17:30–18:00 p.m. At 22:30 p.m., the lights were turned off to maintain the participants’ day-night rhythms. Our experiment was performed at each of four time points: the third day of the pre-BR, the eighth (BR-Mid), the fifteenth day (BR-Late) of the HDBR period, and the fifth day of the post-BR. Participants completed the temporal bisection task with—6° head-down bed rest state in the BR-mid and BR-late phases. Both the pre-BR and post-BR phases were performed in a normal sitting position, with the participants being asked to sit up straight with the viewing angle parallel to the monitor.

**FIGURE 1:**
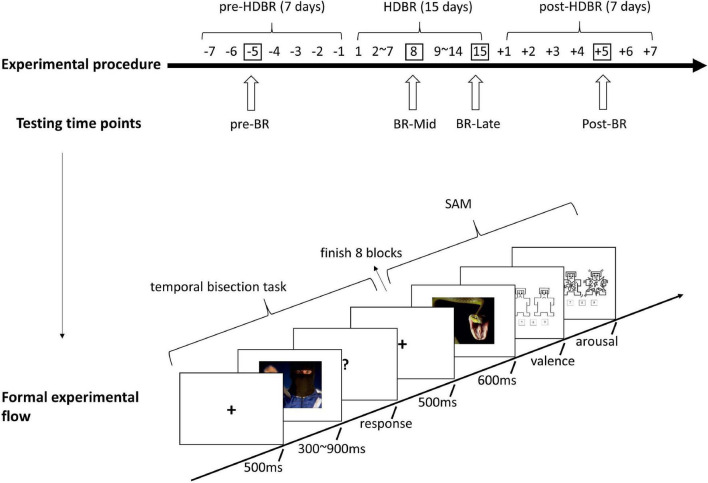
Experimental procedure and formal experimental flow.

Testing was performed using a 13-in monitor (55–75 Hz refresh rate, luminance 250 cd/m2) installed approximately 60 cm apart from the participant. The E-Prime 2.0 software program was used to run the experiment. The experimental session started with the learning phase in which participants were required to memorize two standard durations: 300 ms (short standard duration) and 900 ms (long standard duration). Both standard durations were presented 10 times. During the learning phase, the stimulus used for marking time was a gray rectangle with a size similar to that of the target stimuli. After the learning phase, participants were required to perform eight blocks with three types of emotional pictures: neutral, fear, and disgust. Within each block, three types of pictures were randomly presented for each of the comparison durations (300, 400, 500, 600, 700, 800, 900 ms), for a total of 21 trials. After the presentation of the comparison durations, participants were required to press key “A” if the duration presented was closer to the short standard, or to press the key “L” if the duration presented was closer to the long standard. After the response, there was a 5,000 ms inter-block interval. Between the 5th and 6th blocks, participants can choose whether to rest or not. Besides, after the temporal bisection task, the participants also assessed the 30 pictures in terms of arousal (from very calm to very excited) and valence (from very pleasant to very unpleasant) on the 9-point Self-assessment Manikin scale (SAM) (Bradley and Lang, 1994). The experimental procedure and flow are presented in Figure 1.

### Data Analysis

The statistical analyses were conducted using SPSS 26.0 and MATLAB 2018a. The missing data in the BR-Mid, BR-Late, and post-BR phases (three in total) were handled with mean imputation (Little and Rubin, 2002). As one participant did not have basic time discrimination, as evidenced by a much higher proportion identifying short duration as long duration than long duration in the four temporal bisection tasks, and not due to misremembered response keys, this data was ultimately not included in the analysis. It should be noted that seven of the 16 subjects were taking Chinese herbal medicine during their bed rest (as the Chinese herbal medicine group in the other experiment). The results of the independent samples *t*-test showed no significant difference between the PSE values of these seven subjects and the other nine subjects who did not use medication at the four time points for the three emotional stimuli (all *p* > 0.05), excluding the effect of medication on time perception.

In this study, the proportions of “long” responses were calculated and fitted by a pseudo logistic model (Killeen et al., 1997). Participants’ time perception was measured by two indicators: (1) the point of subjective equality (PSE), that is, the stimulus duration for which participants respond long as often as they do short (percentage of “long” responses = 0.50). A lower PSE value for one stimulus than for another one suggests a lengthening effect (time overestimation effect), with participants responding long more often for the former than for the latter, even though they are of the same duration; (2) the difference limen (DL). It is the half difference in duration between the 25 and 75% points of the logistic function. This is a measure of time sensitivity (time variability): The lower the DL, the greater the sensitivity to time.

## Results

### The Positive Affect and Negative Affect Scores

**FIGURE 2:**
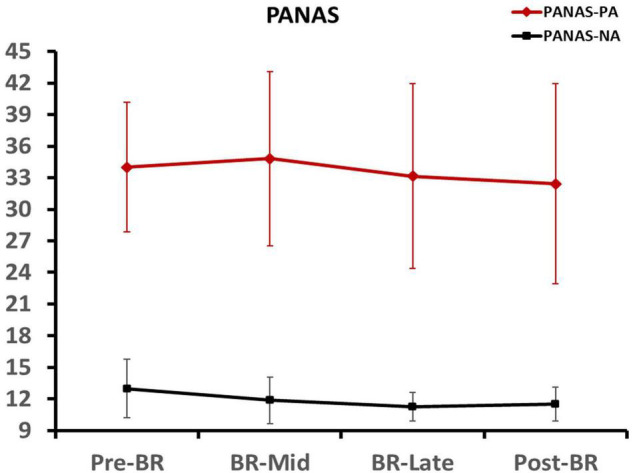
Participants’ positive affect and negative affect scores at different time points. Error bars indicate standard deviation of the mean.

One-Way repeated-ANOVA was used to analyze the participants’ positive affect and negative affect scores at different time points. Results indicated that the effect of time was not significant (all *p* > 0.05), which means participants had the same emotional state before the four tests. These data are presented in Figure 2.

### The Self-Assessment Manikin Scale Rating

A 3 (emotion conditions) × 4 (time points) repeated-measures ANOVA was performed on valence and arousal. For arousal, results indicated that the main effect of time point was not significant [*F*_(3, 45)_ = 2.067, *p* = 0.118, η_*p*_^2^ = 0.121]. For valence, results showed a significant effect of the time point × emotion conditions interaction [*F*_(6, 90)_ = 4.589, *p* = 0.006, η_*p*_^2^ = 0.234], the follow-up Bonferroni test showed that the valance of neutral stimuli was significantly lower in the pre-BR than the BR-Late (*p* = 0.031) and post-BR (*p* = 0.019). That means, although four tests were conducted using the same picture stimuli, except for the valence of the neutral stimuli, the valence and arousal scores of the fear stimuli and disgust stimuli did not change significantly between the four tests.

In four tests, the repeated-measures ANOVA revealed rated valence to differ all significantly among fear, disgust, and neutral stimuli (all *p* < 0.01). The follow-up Bonferroni test indicated that the average valence was lower for disgust stimuli compared to both fear and neutral stimuli (both *p* < 0.01), and the mean valence of fear stimuli was lower than that of neutral stimuli (*p* < 0.01). Furthermore, the independent samples *t*-test showed that the subjective ratings of the three types of stimuli were consistent with the mainland male rating of valence from the IAPS. Unfortunately, although we did a secondary screening of high arousal fear stimuli in the IAPS in the pre-experiment, the results showed that participants rated the arousal of the fear stimuli significantly lower than the mainland male rating of the IAPS.

Means and standard deviations of SAM are presented in Table 1.

**TABLE 1:** The Assessment of Emotions with 9-point Scales SAM (M ± SD).

		IAPS	Pre-BR	BR(Mid)	BR(Late)	Post-BR
Valence	Neutral stimuli	4.17 ± 1.20	3.34 ± 1.92	4.06 ± 1.82	4.43 ± 1.68	4.58 ± 1.66
	Fear stimuli	5.57 ± 1.85	5.61 ± 2.11	5.99 ± 1.89	6.01 ± 1.70	6.13 ± 1.40
	Disgust stimuli	7.03 ± 1.36	7.56 ± 1.76	7.13 ± 1.51	7.16 ± 1.61	7.25 ± 1.42
Arousal	Neutral stimuli	2.86 ± 1.98	2.88 ± 1.87	2.03 ± 1.67	2.19 ± 1.56	2.11 ± 1.73
	Fear stimuli	6.53 ± 2.00	4.52 ± 2.02	4.51 ± 2.32	4.34 ± 2.25	3.90 ± 2.22
	Disgust stimuli	6.65 ± 2.20	5.59 ± 2.45	5.82 ± 2.54	5.48 ± 2.14	5.11 ± 2.32

*The higher the score, the lower the valence and the higher the arousal.*

### Time Perception

#### Point of Subjective Equality

A 3 (emotion conditions) × 4 (time points) repeated-measures ANOVA was performed on PSE. The main effect of emotion conditions was marginally significant [*F*_(1.407, 21.100)_ = 3.616, *p* = 0.058, η_*p*_^2^ = 0.194], but the main effect of time points and the interaction were all not significant. Pairwise comparisons showed that the PSE of fear stimuli was significantly lower than neutral stimuli (*p* = 0.023) and disgust stimuli (*p* = 0.007).

**FIGURE 3:**
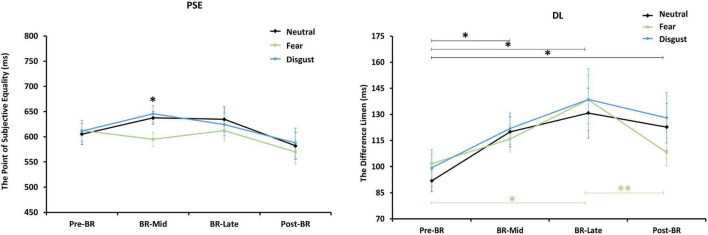
The figure on the left represents the changes of participants’ PSE mean values at different time points. The right represents the changes of participants’ DL mean values at different emotion conditions. Error bars indicate standard errors of the mean. ^**^p < 0.01, *p < 0.05.

**FIGURE 4:**
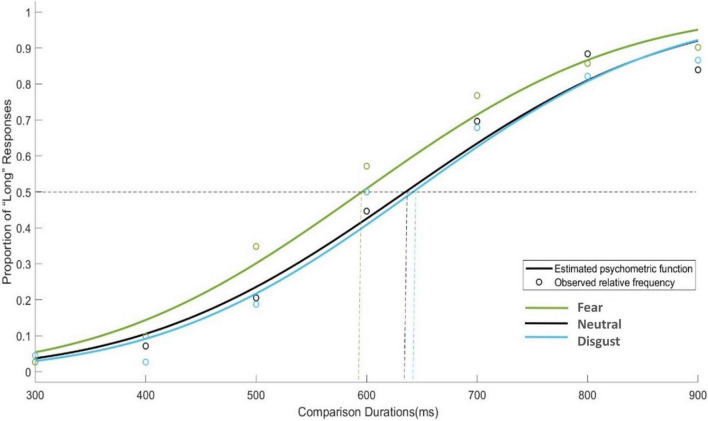
Mean proportions of “Long” responses plotted against probe durations and fitted psychometric functions for each emotional condition in BR phase. The dashed curve represents the PSE values estimated by psychometric function, the circle represents the observed relative frequency.

To further examine the changes in PSE during the bed rest experiment, four one-way ANOVAs with emotion conditions as a factor were performed on PSE respectively. Only in BR-Mid phase, the main effect of emotion conditions was significant [*F*_(2, 45)_ = 4.750, *p* = 0.013]. The trend in PSE showed that although the PSE values for fear stimuli were smaller than those for neutral and disgust stimuli in the BR-Late and post-BR phases, the differences were not significant (see Figure 3). Then, Figure 4 depicts the relative response proportions and the psychometric curves for the neutral, fear and disgust condition in BR-Mid phase, resulting from the Pseudo-Gaussian model (Killeen et al., 1997). The results revealed that both the fitted curve and the PSE for the fear stimuli shifted leftward relative to the neutral and disgust stimuli, indicating that participants overestimated time in BR-Mid phase.

To explore subjects’ estimates of each duration, the proportion of “long” responses for each duration period was used as the dependent variable for analysis. A two-way repeated measurement ANOVA [3 (emotion conditions) × 7 (durations)] in BR-Mid phase was performed. The main effect of durations and emotion conditions and their interaction were significant. Also, three factor repeated measurement analysis of variance [3 (emotion conditions) × 7 (durations) × 4 (time points)] was also performed. The main effect of durations and emotion conditions and their interaction were all significant (the specific statistical results see Supplementary File).

#### Difference Limen

A 3 (emotion conditions) × 4 (time points) repeated-measures ANOVA was performed on DL. The main effect of time points was significant [*F*_(3, 45)_ = 4.089, *p* = 0.012, η_*p*_^2^ = 0.214], but the main effect of emotion conditions and the interaction were all not significant. Pairwise comparisons showed that the DL in pre-BR was significantly lower than BR-Mid (*p* = 0.022), BR-Late (*p* = 0.010), and post-BR (*p* = 0.017), indicating that participants’ time sensitivity became poor in BR phase and post-BR phase.

To further explore changes in DL across emotional stimuli at the four time points, one-way repeated measures ANOVAs with time as the independent variable for DL values for neutral, fear, and disgust, respectively. Results showed that the main effect of neutral stimuli was significant [*F*_(3, 45)_ = 2.648, *p* = 0.06, η_*p*_^2^ = 0.150]. Pairwise comparisons indicated that the DL in pre-BR was significantly lower than BR-Mid (*p* = 0.021), BR-Late (*p* = 0.025), and post-BR (*p* = 0.019). And the main effect of fear stimuli was significant [*F*_(3, 45)_ = 4.504, *p* = 0.008, η_*p*_^2^ = 0.231]. Pairwise comparisons showed that the DL in pre-BR was significantly lower than BR-Late (*p* = 0.015), the DL in post-BR was significantly lower than BR-Late (*p* = 0.008). But the main effect of disgust stimuli was not significant (see Figure 3).

## Discussion

This study aimed to explore the effects of emotional stimuli on time perception and its changes under –6° head-down bed rest, which simulates the physiological response to weightlessness that occurs during space flight.

Compared to pre-BR phase, there was a tendency to overestimate the time of fear stimuli in the BR-Mid, BR-Late, and post-BR phase, particularly in the BR-Mid period, where participants significantly overestimated the fear stimuli compared to neutral and disgust stimuli. As mentioned in the introduction, the internal clock model suggests that arousal affects the rate at which the pacemaker generates pulses. The higher the arousal, the more pulses are accumulated, causing time overestimation (Droit-Volet et al., 2004; Effron et al., 2006). However, we do not seem to be able to attribute the temporal overestimation effect of fear stimuli purely to the effect of the arousal level of emotional stimuli, as there was no difference between the arousal level of fear and disgust stimuli in the BR-Mid phase. The results of the effect of disgust stimuli on time perception are inconsistent, which may vary depending on the source of the emotion. Gil and Droit-Volet (2011) found that the facial expression of disgust does not involve any time distortion. However, in another study it was found that viewing disgust stimuli of food caused time underestimation (Gil et al., 2009). When using stimuli of mutilated body as disgust stimuli, Angrilli et al.’s (1997) study found a time overestimation effect, whereas Shi et al.’s (2012) study did not find time distortion, in line with our results. We suspect that this may be related to the paradigm used. Angrilli used a time reproduction task, while Shi’s study, like us, uses the temporal bisection task. One alternative explanation: disgust was “averting something that is harmful to our health or tastes bad,” as opposed to the fear stimuli to avoid imminent danger and prepare for action (Bradley et al., 2001). In terms of motivation, while fear and disgust both motivate withdrawal, they may do so for different reasons. Fear-motivated avoidance protects the person from perceived danger, while disgust-motivated avoidance may be more often linked to sensation or imagery (Woody and Teachman, 2000). Thus, even if disgust is considered a high arousal emotion, the organism does not need to react to an imminent threat. Individual arousal levels do not increase, nor do they accelerate the internal clock and thus do not cause time distortion.

In fact, participants did not rate arousal to fear stimuli very highly during the BR-Mid phase, which is in line with previous research concluding that HDBR causes individuals emotional blunting when processing negative picture stimuli (Brauns et al., 2019). The overestimation of temporal duration by fear stimuli may be related to the altered body state of participants. It has been shown that when stimuli with specific motor implications produce (potential) behavioral responses that are consistent with the participant’s body state, the corresponding behavioral system can be better activated (Niedenthal, 2007; Ye, 2014). For example, after the disappearance of a rapidly presented sad word or a happy word, the participants was were asked to perform an approach action (flexing the arm) or an avoidance action (extending the arm). The results showed that the approach action was faster than the escape action after the happy word, and vice versa for the sad word (Alexopoulos and Ric, 2007). This interaction between the body and external stimuli to produce a behavioral response can facilitate the perception of emotional stimuli (Cacioppo et al., 1993; Stepper and Strack, 1993; Centerbar and Clore, 2006; Harmon-Jones and Peterson, 2009) and thus influence arousal modulation of temporal perception (Adelmann and Zajonc, 1989). Thus, when presented with fear stimuli, participants develop a behavioral tendency to move away from the stimulus, and the backward leaning body state can be seen as an avoidance action that facilitates the perception of the fear stimuli (Wu et al., 2011). This behavioral imitation consistent with the action implications of emotional stimuli influences time perception. increasing the arousal of the fear stimuli, which in turn affects time perception.

Interestingly, we found that the temporal overestimation effect of fear stimuli disappeared in the BR-Late phase, even when participants remained in the –6° head-down bed rest state. This may be because not only does body state alone affect temporal perception, but also requires participants to be more attentive to body state. Research has shown that awareness of body states influences emotional temporal perception, with participants’ attention to body states enhancing emotional temporal distance distortion (Pollatos et al., 2014). During the bed rest experiment, we did find that in the early stages of bed rest, participants were more aware of their physical state and experienced more pronounced changes in their physiological responses, such as dizziness and lower back pain. In the later BR phase, participants reported that they were completely comfortable in the head-down position and that they “felt that their body state was not different from resting in bed in their daily lives” and that they were less aware of their body state. These are known through psychological interviews and doctor questioning, and although it is relatively subjective, it fits the four-stage model of emotional change in spatial environments. Specifically, the first stage consists of physical discomfort causing psychological discomfort (Gushin et al., 1993). In the early published literature, it is indeed possible to find a reduction in proprioceptive feedback in astronauts in a space environment in microgravity that affects cognitive performance during the initial phase and gradually recovers during the later phases (Semjen et al., 1998). Overall, our results supported to some extent the hypothesis that head-down bed rest prolongs subjective time perception of fear stimuli, suggesting that simulation of emotional stimuli and preparation for action influenced time perception to a greater extent than the single dimension of emotion.

Compared to pre-BR phase, there was a significant decrease in overall temporal sensitivity in the BR-Mid, BR-Late, and post-BR phase. While no statistically significant effect on the DL was observed under three emotion conditions in different phase, consistent with the results of previous studies indicating that emotional stimuli do not distort organisms’ temporal sensitivity (Gil and Droit-Volet, 2011; Fayolle et al., 2015). However, the temporal sensitivity tends to get worse in the BR phase (mid and late) and better in the post-BR phase, especially for neutral and fear stimuli. This change is more likely to be caused by HDBR. Previous physiological studies on HDBR model have found that head-down bed rest leads to reduced vagal excitability, and this effect has been observed more with increasing time spent in HDBR (Crandall et al., 1994; Hirayanagi et al., 2004; Sun et al., 2014), and researchers have demonstrated that lower vagal activity is associated with lower error (means high time sensitivity) in the time bisection task (Cellini et al., 2015). Other possible reasons: as reported in previous studies, the lengthening effect of subjective time due to emotional stimuli disappears during long duration judgments (usually above 4 s). This is because the pacemaker rate increases in response to the emotional event and then gradually return to baseline over time as the emotion decays (Angrilli et al., 1997; Noulhiane et al., 2007). Our results suggested that multiple presentations of short-term stimuli (300–900 ms), even with long intervals between presentations (at least 5 days apart in this study), may also lead to a lack of affective effects and thus a reduction in pacemaker rate. In addition, also in the normal sitting position, there was a tendency for participants to overestimate time for the three types of emotional stimuli in the post-BR phase compared to the pre-BR phase (PSE values were below 600 for all three stimuli), and although this difference was not significant, it is reasonable to suspect that there is also some unknown effect on time perception when the participants’ body state is changed from head-down bedrest to normal sitting, which needs to be further explored in the future.

Our study had some limitations. Firstly, psychophysiological measurements (e.g., heart rate variability, event-related potentials, galvanic skin response) might be useful to more directly assess enhanced arousal when participants are processing the emotion stimuli. Secondly, Due to the lack of comparative data for short periods of -6 degrees head-down bed rest, further research is needed to determine whether there is a potential effect of a third variable other than posture on the change in time perception during bed rest. Lastly, the lack of female participants does not explicitly rule out whether gender is a moderating variable that affects time perception in the –6° head-down bed rest state.

## Conclusion

This study used three emotional picture stimuli: neutral, fear, and disgust, in combination with a temporal bisection task to measure 16 male participants’ time perception in 15 days of –6° head-down bed rest. We found that: participants showed a temporal overestimation effect for fear stimuli compared to neutral and disgust stimuli in the middle phase of bed rest (day 8), and a decreasing trend in temporal sensitivity to the three types of stimuli in the middle and later phases (day 8 and 15), especially for fear and neutral stimuli. These results may be related on the one hand to the type of emotional stimuli, with the head-down position somewhat imitated the action implications of the fear stimuli. On the other hand, it may be related to changes in physiological factors in participants in the bed rest state, such as the body state and reduced vagal excitability. This suggests that the impact of the microgravity environment and the accompanying changes in psychological and physiological factors on time perception should be taken into account to avoid errors in space operations.

## Data Availability Statement

The raw data supporting the conclusions of this article will be made available by the authors, without undue reservation.

## Ethics Statement

The studies involving human participants were reviewed and approved by the Ethics Committee of Space Science and Technology Institute (Shenzhen). The patients/participants provided their written informed consent to participate in this study.

## Author Contributions

YQ, SJ, and YS involved in experimental design. YQ and SJ collected the data. YQ analyzed the data and wrote the manuscript. LC, BW, and YS revised the whole manuscript. XJ, HQ, BX, LC, and BW organized the experiments and provided financial support. All authors contributed to the article and approved the submitted version.

## Conflict of Interest

The authors declare that the research was conducted in the absence of any commercial or financial relationships that could be construed as a potential conflict of interest.

## Publisher’s Note

All claims expressed in this article are solely those of the authors and do not necessarily represent those of their affiliated organizations, or those of the publisher, the editors and the reviewers. Any product that may be evaluated in this article, or claim that may be made by its manufacturer, is not guaranteed or endorsed by the publisher.
