# Supplementary material for: Effects of 15-Day Head-Down Bed Rest on Emotional Time Perception
Source: Front Psychol. 2021 Dec 22;12:732362. doi: 10.3389/fpsyg.2021.732362 (PMC8727352; doi:10.3389/fpsyg.2021.732362)
Supplement: Supplementary file 1 [file Data_Sheet_1.PDF]

Supplementary file

To explore subjects' estimates of each duration, the proportion of "long" response for each duration period is used as the dependent variable. A two-way repeated measurement ANOVA [3(emotion conditions) × 7 (durations)] in BR-Mid phase was performed. The main effect of durations ($F_{(3.667,55.010)}$ = 367.219, p = 0.000, η_p^2=0.961) and emotion conditions ($F_{(2,30)}$ = 13.705, p = 0.000, η_p^2=0.477) were all significant, and the interaction was also significant ($F_{(5.165,77.478)}$ = 2.517, p = 0.035, η_p^2=0.144). The results of the simple effects analysis showed that the fear stimuli differed significantly from the other two types of stimuli from the duration of 400ms (the proportion "long" response of fear stimuli was higher than neutral stimuli at 500ms (p=0.008), 600ms (p=0.019), 900ms (p=0.04), and the proportion "long" response of fear stimuli was higher than disgust stimuli at 400ms (p=0.013), 500ms (p=0.002), 600ms (p=0.047), 700ms (p=0.017)).

Three factor repeated measurement analysis of variance [3(emotion conditions) × 7 (durations) ×4 (time points)] was also performed. The main effect of durations ($F_{(2.757,41.359)}$ = 546.185, p = 0.000, η_p^2=0.973) and emotion conditions ($F_{(1.448,21.713)}$ = 3.815, p = 0.05, η_p^2=0.203) were all significant, and the interaction between emotion conditions and durations was also significant ($F_{(12,180)}$ = 2.478, p = 0.005, η_p^2=0.142). The results of the simple effects analysis showed that the proportion "long" response of fear stimuli was higher than disgusting stimuli at 500ms (p=0.001), and the proportion "long" response of disgust stimuli was higher than neutral stimuli at 300ms (p=0.029) (these curves see figure 1 below).

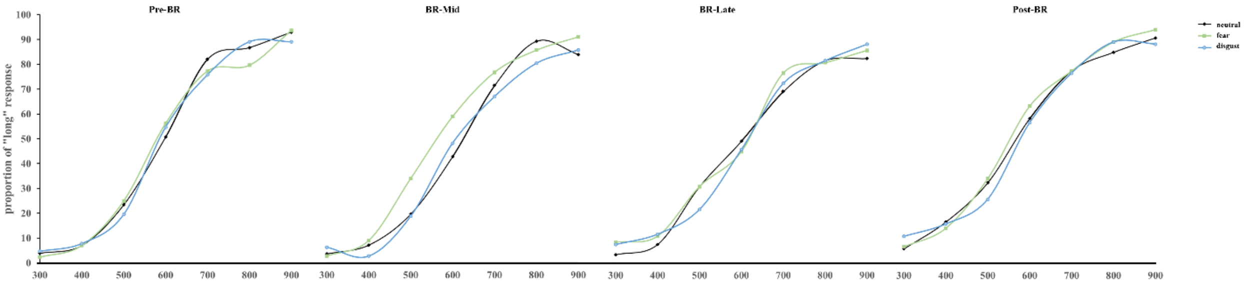

Supplementary Figure 1. Mean proportions of "Long" responses for each duration period at different time points.
